# Involvement of Polycomb Repressive Complex 2 in Maturation of Induced Pluripotent Stem Cells during Reprogramming of Mouse and Human Fibroblasts

**DOI:** 10.1371/journal.pone.0150518

**Published:** 2016-03-03

**Authors:** Niusha Khazaie, Mohammad Massumi, Ping Wee, Mahdieh Salimi, Abdulshakour Mohammadnia, Moein Yaqubi

**Affiliations:** 1 Institute of Medical Biotechnology, National Institute of Genetic Engineering and Biotechnology (NIGEB), Tehran, Iran; 2 Department of Physiology, University of Toronto, Toronto, ON, Canada; 3 Lunenfeld-Tanenbaum Research Institute, Mount Sinai Hospital, Toronto, ON, Canada; 4 Department of Medical Genetics and Signal Transduction Research Group, Faculty of Medicine and Dentistry, University of Alberta, Edmonton, AB, Canada; Georgia Regents University, UNITED STATES

## Abstract

Induced pluripotent stem cells (iPSCs) provide a reliable source for the study of regenerative medicine, drug discovery, and developmental biology. Despite extensive studies on the reprogramming of mouse and human fibroblasts into iPSCs, the efficiency of reprogramming is still low. Here, we used a bioinformatics and systems biology approach to study the two gene regulatory waves governing the reprogramming of mouse and human fibroblasts into iPSCs. Our results revealed that the maturation phase of reprogramming was regulated by a more complex regulatory network of transcription factors compared to the initiation phase. Interestingly, in addition to pluripotency factors, the polycomb repressive complex 2 (PRC2) members Ezh2, Eed, Jarid2, Mtf2, and Suz12 are crucially recruited during the maturation phase of reprogramming. Moreover, we found that during the maturation phase of reprogramming, pluripotency factors, via the expression and induction of PRC2 complex members, could silence the lineage-specific gene expression program and maintain a ground state of pluripotency in human and mouse naïve iPSCs. The findings obtained here provide us a better understanding of the gene regulatory network (GRN) that governs reprogramming, and the maintenance of the naïve state of iPSCs.

## Introduction

A wide range of mouse and human somatic cells can acquire pluripotency characteristics by using a defined set of four transcription factors (TFs), including Oct4/Pou5f1, Sox2, Klf4, and Myc [1,2]. Reprogrammed induced pluripotent stem cells (iPSCs) provide reliable sources for regenerative medicine, but the major challenge is the low efficiency of reprogramming, a problem which remains to be solved [3]. During the reprogramming of mouse and human fibroblasts, cells undergo two dramatic changes in their gene expression profiles [3–5]. The first significant change in expression profile occurs during the initiation phase of reprogramming, when these changes are unstable and can be reversed when Oct4, Sox2, Klf4, and Myc are removed [3,4]. Most fibroblast-initiating reprogramming processes fail to generate mature iPSCs because of cell death or their reversion to initial state prior to reaching the maturation phase [3], which encompasses the second period of significant change in expression profile. This implies that the maturation of iPSCs during reprogramming is much more complex than the initiation phase, and that it needs to be regulated by a variety of TFs.

Although Oct4, Sox2, Klf4, and c-Myc are usually used as the gold standard TFs for reprogramming somatic cells into iPSCs, some other TFs could replace them. For instance, it has been shown that the combination of Bmi1 and Oct4 can successfully reprogram fibroblasts into iPSCs [6]. Besides Bmi1, the DNA hydroxylase Tet1, which can activate the expression of Oct4, could replace Oct4 and induce pluripotency [7]. Also, this factor can be used instead of Oct4 in combination with Sox2, Klf4, and Myc during reprogramming [7]. Moreover, several other regulators, including the polycomb repressive complex 2 (PRC2) [8,9], Zic3 [10], and Rcor2 [11] have demonstrated to be reprogramming-inducing factors that could increase the efficiency of reprogramming. Despite a wide range of studies aiming to increase the efficiency of reprogramming or to identify new TFs that regulate this process, to the best of our knowledge there is no comprehensive study to understand the gene regulation of the cells during reprogramming, especially during the maturation phase of iPSCs.

Gene Regulatory Network (GRN) was plotted and analyzed using powerful bioinformatics approaches and systems biology tools which can efficiently provide precise predictions about the behavior of TFs during reprogramming, during the direct conversion of somatic cells, and during the differentiation of pluripotent stem cells [12,13]. Previously, powerful computational approaches have been used to identify the main TFs involved in reprogramming and direct reprogramming events [12,13]. More recently, the CellNet approach was proposed to compare generated cells with their *in vivo* counterparts based on their gene expression profiles, with an aim for increasing the efficiency of differentiation [14]. In our previous study, we used the gene expression profile data obtained from microarray data and applied a bioinformatics approach to understand the behavior of TFs during the direct conversion of mouse fibroblasts into induced neural stem cells [15]. In spite of powerful techniques and the large amount of highly qualified and high throughput data related to iPSC reprogramming, there is currently no comprehensive study on the role of regulatory factors during the maturation of iPSCs from mouse and human fibroblasts.

In the present study, we investigated the GRNs underlying the initiation and maturation phases of mouse and human fibroblast reprogramming. To that end, six independent and highly qualified microarray expression data sets were analyzed using bioinformatics approaches to understand the regulatory role of TFs during the initiation and maturation phases of reprogramming. Our results showed that the regulation of the gene expression program in the maturation stage is much more complex in comparison to the initiation stage. In addition to pluripotency factors, for the first time, using systems biology and bioinformatics approaches we characterized the role of PRC2 members including Ezh2, Eed, Jarid2, Mtf2, and Suz12 during the maturation phase of reprogramming. The maturation phase of reprogramming has come to be known as the “major road block” to reprogramming. Here, we provided molecular details regarding the regulatory network that underlie the control of the gene expression program during this maturation phase of reprogramming. We believe that the “road map” depicted here can address some issues related to the low efficiency of reprogramming and can be used to improve our understanding of the maturation of reprogrammed cells.

## Materials and Methods

### Microarray availability and analysis

Microarray data sets for the conversion of mouse and human fibroblasts into iPSCs were obtained from Gene Expression Omnibus (GEO) using GSE42379, GSE21757, GSE47489, GSE18226, GSE14897, and GSE34309 accession numbers [3–5,16–18] (Table 1). These six data sets were independently generated using different protocols. Reprogramming into iPSCs was confirmed in these studies by the presence of gene markers and dramatic changes in expression profiles. For four data sets, including Affymetrix Mouse Genome 430A 2.0 Array, Affymetrix Human Genome U133 Plus 2.0 Array, and Affymetrix Human Genome U133A 2.0 Array, normalizations of raw data sets were done using the Robust Multiple-array average (RMA) algorithm, which is available in Flexarray [19]. The normalized data of other Chip types were loaded onto Flexarray. To detect differentially expressed genes (DEGs), the fold change algorithm from the Flexarray software was applied to the data. The *p-values* were calculated for all six data sets and genes with *p-values* less than 0.05 were considered as DEGs.

**Table 1:** Microarray data sets used in this study and their experimental design.

Experiment	Comparison	Accession number	Chip type	Stage
Polo et al., (2012)	Day 6 after induction_SSEA1+ versus KH2-MEF	GSE42379	Affymetrix Mouse Genome 430A 2.0 Array	Initiation
Polo et al., (2012)	Day12 after induction_GFP+ versus Day 6 after induction_SSEA1+	GSE42379	Affymetrix Mouse Genome 430A 2.0 Array	Maturation
Polo et al., (2012)	Day12 after induction_GFP+ versus KH2-MEF	GSE42379	Affymetrix Mouse Genome 430A 2.0 Array	Throughout reprogramming*
Samavarchi-Tehrani et al., (2010)	Day 5 after induction versus MEFs	GSE21757	Affymetrix Mouse Exon 1.0 ST Array	Initiation
Samavarchi-Tehrani et al., (2010)	Day 21 after induction versus Day 5 after induction	GSE21757	Affymetrix Mouse Exon 1.0 ST Array	Maturation
Samavarchi-Tehrani et al., (2010)	Day 21 after induction versus MEFs	GSE21757	Affymetrix Mouse Exon 1.0 ST Array	Throughout reprogramming
Tanabe et al.,(2013)	HDF- Day 28 after induction versus HDF- Day 7 after induction (TRA-1-60(+)	GSE47489	Agilent-028004 SurePrint G3 Human GE 8x60K Microarray	Maturation
Tanabe et al.,(2013)	HDF- Day 28 after induction versus HDF	GSE47489	Agilent-028004 SurePrint G3 Human GE 8x60K Microarray	Throughout reprogramming
Doi et al.,(2009)	Induced pluripotent stem cells versus	GSE18226	Affymetrix Human Genome U133 Plus 2.0 Array	Throughout reprogramming
Si-Tayeb et al., (2010)	Induced pluripotent stem cells versus Human foreskin fibroblasts	GSE14897	Affymetrix Human Genome U133 Plus 2.0 Array	Throughout reprogramming
Wang et al.,(2012)	Induced pluripotent stem cells versus Human dermal fibroblasts	GSE34309	Affymetrix Human Genome U133A 2.0 Array	Throughout reprogramming

*this comparison revealed a list of DEGs in which their expression continually increased or decreased during reprogramming or their expression significantly changed at specific stages and remained at high or low levels in the next stages.

### Functional annotation clustering of DEGs

In order to identify significantly altered biological processes during the reprogramming of mouse fibroblasts into iPSCs, the list of DEGs was submitted to the Database for Annotation, Visualization and Integrated Discovery (DAVID) [20,21]. This database identifies clusters of genes based on their biological function in functional annotation clustering analysis. Each cluster has a specific enrichment score; in our study an enrichment score higher than 1.3 (*p-value*<0.05) was accepted as a significantly altered biological process.

### Identification of transcription factor binding sites

To identify regulators of DEGs involved during the reprogramming of mouse fibroblasts into iPSCs, we used experimentally based databases, including ChIP Enrichment Analysis (ChEA) and TFacts [22,23]. The ChEA database contains results from high throughput data regarding transcription factor binding sites. This database contains 458,471 regulatory interactions for more than 200 TFs, and provides the opportunity to analyze several TFs from one list at the same time. Results extracted from the ChEA database were filtered based on the *p-value* and their expression. TFs with *p-value*<0.05 were selected. Based on the importance of TFs in gene regulation, we selected TFs with a fold change of more than 1.5 as differentially expressed (DE)-TFs.

### Construction of TF protein-protein interaction network and analysis of protein complexes

Experimentally validated and predicted interactions were retrieved from BioGRID and STRING databases [24,25]. Currently, BioGRID has deposited more than 796,767 protein and genetics interactions from more than 50,000 publications. Experimentally validated protein-protein interactions for DE-TFs retrieved from BioGRID were filtered by expression data. In addition to BioGRID, we used the STRING database to enrich our data with more protein-protein interactions. We downloaded data with high confidence scores (0.7) and applied expression data on them to identify precise interactions.

Protein-protein interactions for DE-TFs were combined with expression data and visualized in Cytoscape software [26]. Constructed networks were subjected to protein complexes analysis using the MCODE plugin of Cytoscape. The most significant protein complexes in the constructed network were identified by the cut-off degree set at 2.

### Gene Regulatory Network (GRN) construction and analysis

To construct the GRN during reprogramming of mouse fibroblasts into iPSCs, different sources of data, including TF binding sites, TF protein-protein interactions, and expression data were integrated and visualized in Cytoscape. Constructed networks were subjected to gene ontology and centrality analyzes. To identify the most affected biological processes in the constructed regulatory networks, the ClueGO and CluePedia plugins of Cytoscape were used [27,28]. In the advanced statistical option of the tools, Two-sided hypergeometric test was selected to calculate the importance of each term and Bonferroni step-down was used for *p-value* correction.

In order to find the hubs of each constructed network, we used the degree parameter. Degree is the simplest connectivity index, which considers the neighbors of each gene. We used out-degree to explore the most important regulator and in-degree to find the most regulated genes. To achieve this aim, the CentiScaPe plugin of Cytoscape was used [29].

### Clustering gene expression data

Clustering of DEGs was done using Gene Cluster software and visualized using Java TreeView. We used hierarchical clustering which is a strong method for analyzing high throughput expression data. Similarity metrics were calculated for both genes and arrays. To measure the similarity of both arrays and genes, correlation (uncentered) was used [30,31].

## Results

During the reprogramming process, cells experience two dramatic changes in their gene expression profile. The first change occurs during the initiation stage and the next one during the maturation stage of iPSCs. In this investigation, our comprehensive analysis of gene expression profiles revealed more changes in the maturation stage compared to the initiation stage. In the following parts of this study, we mainly focused on the maturation of reprogrammed cells and on the identification of the main regulators of this stage.

### Initiation of mouse embryonic fibroblasts reprogramming into induced pluripotent stem cells

By comparing the gene expression profile of cells in the initial stage of reprogramming with that of mouse embryonic fibroblasts (MEFs) from two studies (Polo et al., and Samavarchi-Tehrani et al.), 124 DEGs with the same expression pattern in both studies were identified [4,5] (S1 Table). In this list, 90 genes were up-regulated whereas 34 genes were down-regulated. DEGs were categorized in different terms based on their function, including signaling role, endopeptidase inhibitor activity, cell adhesion contribution, and cytoskeletal function. In the up-regulated gene list, we found genes involved in mesenchymal-to-epithelial transition (MET), including *Cdh1*, *Cldn11*, and *Epcam* had significantly increased expression during the initiation phase of reprogramming. At the same time, however, genes including *Col7a1*, *Gjb2*, *Il11*, *Itga5*, Olr1, *Smoc2*, *Tgfb1i1*, and *Wisp1*, which are involved in cell adhesion and cell-cell contacts, were significantly down-regulated during this stage.

To understand the contribution of the TFs involved in gene expression regulation, a GRN was constructed for DEGs. This network harbored 113 DEGs and Differentially Expressed-TFs (DE-TFs) that connected to each other by 254 regulatory and protein-protein (PPI) interactions. Collectively, eight up-regulated TFs were identified to be involved in the regulation of the gene expression program during the initiation of reprogramming. Among them, pluripotency factors including *Nanog*, *c-Myc*, and *Sall4* were detected, but two markers of maturation, namely *Oct4* and *Sox2*, were not found. Interestingly, our data showed that during the transition between MEFs and initiation stage cells, the TFs *Suz12* and *Mtf2*, which are members of PRC2, played roles as main regulators of the transition. Despite the presence of these two factors, the core catalytic protein of PRC2, *Ezh2* and other associated members were not found as regulators. In this network *Nanog*, *Suz12* and *Mtf2* were found to be top three hub genes. In addition, some genes such as *Sall4*, *Etv5*, *Cdh13*, and *Zfp704* were highly regulated by the profile of TFs in this transition.

Finally, to reveal the role of DE-TFs on the regulation of DEGs, we studied the regulatory interactions between DE-TFs. The constructed GRN showed that *Nanog* is a main regulator amongst other TFs and that *Sall4* and *Mtf2* are the most regulated TFs.

### Regulation of the transition from initiation to maturation stage by a complex network of transcription factors

Comparing the results between the initiation stage and the maturation stage revealed more complexity in the regulation of the gene expression program during the maturation of iPSCs from MEFs. To provide a better insight on gene expression regulation, we also compared the expression profiles of mature iPSCs with the expression profiles of MEFs in addition to comparing mature iPSCs with initiation stage cells during the reprogramming procedure. This comparison led to the identification of genes in which their expression levels gradually but constantly increased or decreased during the reprogramming stages. Furthermore, this comparison allowed us to obtain a list of genes in which their expressions were significantly changed at a certain stage but remained constant until the maturation of iPSCs. We performed four different comparisons based on two datasets in two categories. In the first category, we compared the expression profiles of the transition from initiation to maturation stage, and in the second category, we compared the expression profile of mature cells with MEFs. To call a gene a DEG, its expression should be changed significantly in both data sets in at least one of the comparisons (i.e. in the comparison of initiation stage with maturation stage or in the comparison of MEFs with cells in the maturation stage). Beside significant changes in expression, the genes should have the same pattern in both examinations. Collectively, we identified 356 DEGs based on the criteria (S2 Table). In the first view, the numbers of DEGs in the maturation phase were dramatically higher than those that appeared in the initiation phase. In this list, during the maturation phase, 230 genes were down-regulated whereas 126 genes were up-regulated (S2 Table).

The down-regulated genes are involved in dozens of biological events, including cell migration, regulation of locomotion, and regulation of cell motility. On the contrary, the up-regulated genes were mostly involved in stem cells and developmental properties, such as stemness maintenance, negative regulation of cell adhesion, and gastrulation processes. The most prominent characteristics of cells based on the results are that during the transition from initiation stage to maturation stage, cells acquired pluripotency characteristics and lost their fibroblast gene expression program. To obtain a deeper insight into the regulatory events that lead to this outcome, regulators of 356 DEGs were found using high throughput data. Interestingly, we identified 41 DE-TFs that have roles in the maturation of iPSCs from MEFs. This list was substantially more complex than the initiation stage of reprogramming. In this list, nearly all critical pluripotency factors, including *Oct4*, *Sox2*, *Nanog*, *Myc*, and *Sall4* were found. In addition to pluripotency factors, all members of PRC2, including *Ezh2*, *Eed*, *Suz12*, *Mtf2*, and *Jarid2* were detected. In order to clarify the importance and exact roles of these 41 DE-TFs, the constructed network was analyzed to identify the most affected processes, hub genes, protein complexes, and finally the regulatory relationship between these DE-TFs.

**Fig 1:**
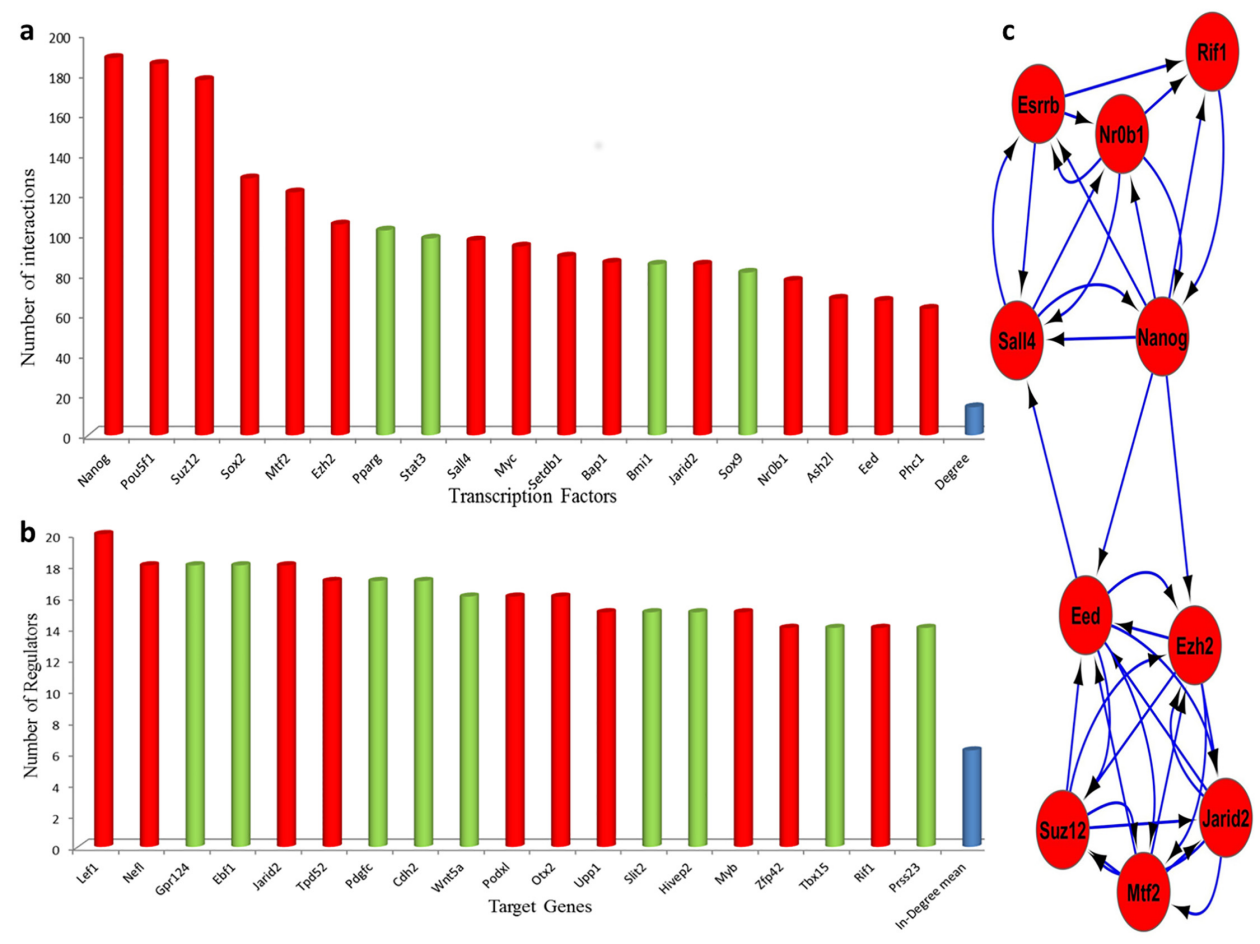
Centrality and protein complexes analyses during maturation of reprogramming. (a & b) Centrality analysis of the network was done using degree and In-degree parameters for ranking the central regulators of genes in the network and the most regulated genes respectively. The red column shows up-regulation whereas the green column shows down-regulation of genes. (c) Protein complexes analysis using valid protein-protein interactions. The red color shows up-regulation and arrows indicate the direction of binding.

TF binding sites were integrated with protein-protein interaction and expression data to construct a GRN for these 356 DEGs (S3 Table). This network accommodated 372 genes, which connected to each other by 2591 interactions (S3 Table). As expected, pluripotency factors, including *Nanog*, *Oct4*, *Sox2*, *Sall4*, and *Myc* were indwelled among the top fifteen regulators of the network. In addition to pluripotency factors, four members of PRC2, including *Suz12*, *Mtf2*, *Ezh2*, and *Jarid2* were found between these fifteen regulators (Fig 1A). Another member of this complex, *Eed* also ranked as the eighteenth regulator in the constructed GRN (Fig 1A). Beside dissection of the integrated regulatory networks, we investigated regulatory interactions to identify the most important regulators. Out-degree analysis revealed *Suz12* as the main regulator of the DEGs with 165 targets (S1 Fig). *Nanog*, *Oct4*, *Mtf2*, and *Sox2* ranked as top regulators of the DEGs following *Suz12*. The results highlight the importance of the PRC2 complex in addition to pluripotency factors during the maturation of iPSCs from MEFs (S1 Fig). Finally, we performed in-degree analysis to identify the most regulated DEGs. Based on this analysis, *Lef1* was identified to be regulated by 20 different DE-TFs (Fig 1B). *Lef1* is known to be involved in developmental processes. Furthermore, our ontology results showed the contribution of *Lef1* in the negative regulation of cell adhesion, formation of primary germ layer, gastrulation, and many other related processes.

These results showed that PRC2 members in collaboration with pluripotency factors play important roles as central regulators in the maturation of iPSCs. In the next step, we investigated the protein-protein interaction networks of DE-TFs to identify potential protein complexes. To increase the validity of predicted protein complexes, experimentally validated protein-protein interactions were used. Interestingly, constructed protein-protein interaction networks revealed two significant protein complexes. The first one accommodated all five members of PRC2, including EZH2, EED, JARID2, MTF2, and SUZ12, along with other proteins including, NANOG, SALL4, ESRRB, NR0B1, and RIF1 (Fig 1C). The second significant protein complex was composed of two main pluripotency factors OCT4 and SOX2, which interacted with Parp1 (S2 Fig). In summary, protein complexes analysis revealed that PRC2 members made up a protein complex to regulate gene expression during maturation of reprogramming.

**Fig 2:**
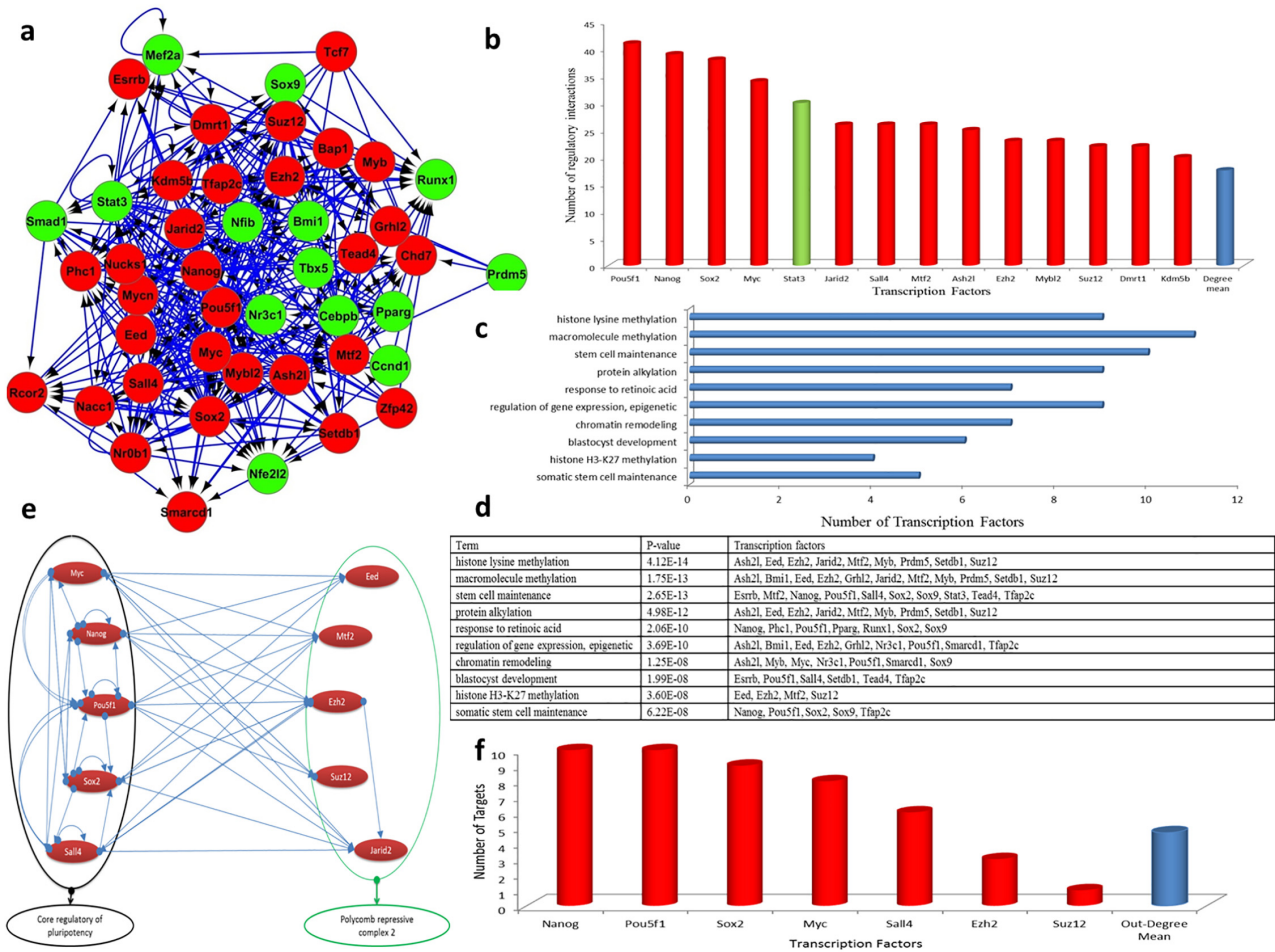
Core regulatory network for DE-TFs during maturation of reprogramming. (a) The core regulatory network between differentially expressed regulators. Red and green colors show up- and down-regulation respectively. (b) Centrality analysis of core regulators network. (c & d) show the most affected processes and regulators involved in these processes. (e & f) The regulatory network between pluripotency factors and PRC2 members during maturation was constructed and subjected to out-degree analysis.

**Fig 3:**
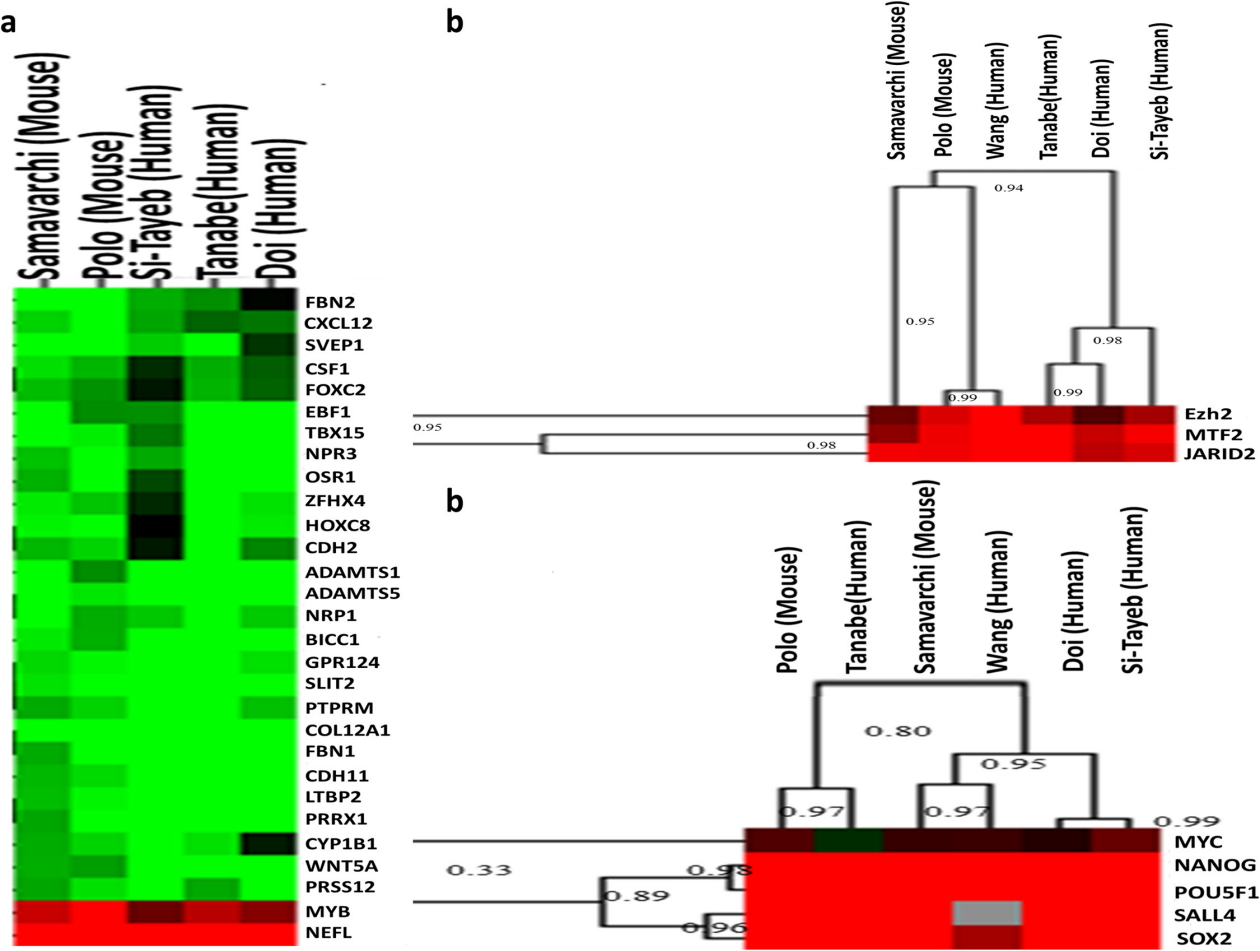
Clustering analysis of PRC2 targets in both mice and humans. (a) Clustering of common targets of PRC2 members in both mice and humans. Red and green colors indicate up- and down-regulation respectively. (b) Ezh2, Mtf2, and Jarid2 gene expression correlation between mice and humans using six different data sets. (c) Gene expression correlation between pluripotency factors in both mice and humans across six independent data sets.

Hitherto, we found that PRC2 and stem cell factors are involved in the regulation of the maturation stage of reprogramming. The PRC2 members showed high numbers of targets and similar expression patterns, therefore they should play a significant role in the composition of the protein complex. To understand the regulatory relationship between the TFs and affected processes, the core regulatory network was constructed (Fig 2A). The core regulatory network was more complicated than the network constructed based on the regulatory interactions of 356 DEGs (S2 Table). For example, each gene was regulated by 6.16 regulators in the constructed network for 356 DEGs, but each DE-TF was regulated by 8.93 regulators in the core regulatory network. Such differences showed that the expression of TFs involved in the reprogramming was more strictly regulated in comparison to other DEGs. Centrality analysis of the core regulatory network revealed that pluripotency factors play a major role in the regulation of DE-TF gene expression; for instance *Pou5f1*, *Nanog*, *Sox2*, and *Myc* were the top four regulators amongst other regulators (Fig 2B). However, four members of PRC2 including *Ezh2*, *Jarid2*, *Mtf2*, and *Suz12*, appeared in the top fifteen regulators and played a crucial role in the core regulatory network (Fig 2B). Finally, this network was analyzed to identify the most affected biological processes during reprogramming. Interestingly, our analysis showed that the most affected processes could be categorized into two main groups (Fig 2C and 2D): first, the processes of methylation and epigenetics changes, and second, the processes involved in stem cell maintenance and development. The most affected process controlled by DE-TFs was histone lysine methylation which leads to the down-regulation of gene expression. The genes *Ash2l*, *Eed*, *Ezh2*, *Jarid2*, *Mtf2*, *Myb*, *Prdm5*, *Setdb1*, and *Suz12*, involved in the methylation of lysine residues, were modulated. The majority of DEGs were down-regulated during the transition from initiation to maturation of reprogramming (230 were down-regulated whereas 126 genes were up-regulated). To understand the actual role of PRC2 members on epigenetic gene expression regulation, we considered 45 common target genes which were controlled by *Ezh2*, *Jarid2*, and *Mtf2* TFs (Fig 3A). Interestingly, 91 percent of those targets were significantly down-regulated during the maturation of iPSCs from MEFs (Fig 3A). In this list, based on the ontology analysis, we found 13 genes which were involved in the process of cell migration. Thus, it seems that PRC2 members are involved in the down-regulation of genes that are highly expressed in MEFs. Besides, the ontology results of the down-regulated genes identified cell migration processes as the most regulated process, having the most numbers of affected genes and lowest *p-value*. Collectively, these results suggest a crucial role of PRC2 in the down-regulation of fibroblast-specific genes, which promote the generation of iPSCs through the maturation stage. The second most affected feature controlled by DE-TFs was stem cell properties. For example, *Esrrb*, *Mtf2*, *Nanog*, *Oct4*, *Sall4*, *Sox2*, *Sox9*, *Stat3*, *Tead4*, and *Tfap2c*, which are involved in stemness maintenance, were modulated. Interestingly, ontology of 126 up-regulated genes showed that the genes were mainly involved in stemness maintenance and developmental processes. In the list, 72 DEGs which are common target genes for three pluripotency factors *Oct4*, *Sox2*, and *Nanog* were identified. Interestingly, a large part of these DEGs, including 56 out of 72 DEGs, were up-regulated (S4 Table). Hence, high throughput data analyses showed that up-regulated genes mainly had the same expression pattern or co-expressed with regulators of pluripotency.

In conclusion, our results from our comparison of gene expression profiles and from analyzing constructed networks revealed the involvement of the PRC2 complex beside pluripotency factors during the maturation stage of iPSCs from MEFs. Our analyses suggested that PRC2 members are involved in the down-regulation of fibroblast-specific genes, whereas pluripotency genes are involved in the up-regulation of stem cell specific genes. In the next step, we tried to predict the role of PRC2 during the reprogramming of human fibroblasts into human induced pluripotent stem cells (hiPSCs).

### Involvement of PRC2 during the reprogramming of hiPSCs from human fibroblasts

The involvement of PRC2 during the maturation of mouse iPSCs from MEFs may suggest that a human homologue complex may perform the same function during reprogramming. To examine this, the expression data set from Tanabe et al. (2013), was used [3]. This data set includes the data of both the initiation and maturation stages of the reprogramming process. Surprisingly, the contribution of PRC2 in the regulation of DEGs during the maturation of human fibroblast reprogramming was not detected. Our investigation showed that the ChEA database, which was used as the source of high throughput data sets for finding regulators of DEGs, did not have human ChIP data for *EED*, *JARID2*, *MTF2*, and *SUZ12*. For *EZH2*, only 40 interactions from one study were retrieved, which was not significant for our comprehensive analysis. To evaluate the potential roles of PRC2 during the maturation of reprogramming in the initiation stage, we sought to find the expression of *EZH2*, *EED*, *JARID2*, *MTF2*, and *SUZ12* TFs in the Tanabe et al., data set. In both the comparison of expression profile of initiation stage cells with maturation stage cells, as well as the comparison of mature cell expression profile with human fibroblast expression profile, we found that the expression of three members of PRC2 including *EZH2*, *JARID2*, and *MTF2* were increased. To increase the validity of these results, three additional data sets that were generated independently and in different laboratories were analyzed (Table 1). Interestingly, in all of the three data sets, *EZH2*, *JARID2*, and *MTF2* were up-regulated when the expression profiles of iPSCs were compared with human fibroblasts (Fig 3B).

The gene expression pattern of pluripotency factors in both mouse and human data sets were clustered (Fig 3C). The clustering results showed a high correlation between the expression of these factors in all six mouse and human data sets. These correlations were more significant between *OCT4*, *NANOG*, *SOX2*, and *SALL4* (Fig 3C). This analysis was used as the standard to compare the expression patterns of *EZH2*, *JARID2*, and *MTF2* in both mouse and human data sets. As we expected, these three factors showed a high correlation in gene expression in all six microarray data sets (Fig 3B). The correlation between *EZH2*, *JARID2*, and *MTF2* was even higher than the correlation between *OCT4*, *NANOG*, *SOX2*, and *SALL4*. These results suggested the importance of the expression of these factors in the reprogramming of human fibroblasts to hiPSCs. To dissect the gene expression regulation exerted by the PRC2 members during human fibroblast reprogramming to hiPSCs, we found common targets of Ezh2, Jarid2, and Mtf2 in mouse data sets. Collectively, we found 45 DEGs to be common targets of these factors, and interestingly 43 of them were differentially expressed in studies by Tanabe et al., (2013), Doi et al., (2009), and Si-Tayeb et al., (2010) [3,4,16] (Fig 1A). We excluded the Wang et al., (2012) study [18], because the microarray chip type they used in their experiment did not contain expression data for a number of genes which we considered as PRC2 targets. In this regard, it could be possible that we lost a number of genes in which their expressions fit well between mouse and human. Interestingly, 29 out of the 43 DEGs were expressed in the same pattern in all five of the data sets for humans and mice (Fig 1A). Even with consideration of the data set from the Wang’s et al., (2012) study [18], 72 percent of the 29 DEGs showed the same expression patterns in at least five or six different data sets. Our analysis during maturation of iPSCs showed that PRC2 members are mainly involved in methylation and repression of gene expression during reprogramming. We sought to examine the expression of 29 identified DEGs in both human and mouse data sets. The expression analysis revealed that 27 out of 29 DEGs were down-regulated and we found over-expression in only two target genes.

Despite the lack of high throughput data sets for binding sites of all human PRC2 members in the sources we used in this investigation, there are high correlations in expression among three PRC2 members, EZH2, JARID2, and MTF2, in both human and mouse data sets (Fig 3B). In addition, the major parts of the PRC2 targets in humans show the same down-regulated expression patterns in mouse data sets. For this reason, we comprehensively used six high quality microarray data sets together with systems biology analyses to show the involvement of PRC2 in the maturation stage of fibroblast reprogramming into iPSCs.

## Discussion

In the current study, the gene expression profiles of both mouse and human fibroblasts undergoing reprogramming into iPSCs were dissected. The results showed that in addition to pluripotency factors, maturation of reprogramming was also regulated by PRC2 members, including *Ezh2*, *Eed*, *Jarid2*, *Mtf2*, *and Suz12*. Interestingly, the integration of gene expression data, gene regulation information, and gene ontology results showed that PRC2 was mainly involved in the repression of fibroblast-specific genes through tri-methylation of H3K27, whereas pluripotency factors up-regulated stem cell-specific genes. The same results were identified specially for EZH2, JARID2, and MTF2 during the maturation stage of human fibroblast reprogramming into hiPSCs.

Our results showed that the early events of reprogramming are less stable and complicated than at the final stages. Low overlapping rates between DEGs in data sets, which were used for the analysis of the early events of reprogramming in comparison with late events, showed that significant variation occurs among cell populations during the first days of reprogramming. The same observation was previously reported, where it was observed that different cells have significant differences in gene expression profiles during the initiation of reprogramming [32]. In contrast to the high variation in gene expression patterns during the initiation stage of reprogramming, there were more similarities in the gene expression profiles of reprogrammed cells during the maturation phase, implying the presence of a common core gene regulatory network governing the maturation of iPSCs. The core regulatory network of DE-TFs involved in maturation revealed two major groups of TFs. The first group was involved in the methylation of H3K27, which is mainly involved in the repression of gene expression, and the second group was involved in pluripotency-related genes. It has been shown that methylation of H3K27 is not highly required for the initiation of reprogramming [33]. In contrast, it has been documented that significant changes in DNA methylation occur in the maturation phase of reprogramming [34]. Clearly, overlapping the different data sets showed that down-regulation of most genes occurred late during reprogramming and that this down-regulation was mainly controlled by PRC2 members. So, inconsistent to previous reports, it seems that PRC2 is a main player for the stable silencing of fibroblast-specific and development-related genes.

Previous reports have clearly demonstrated the significant roles of different members of PRC2 in pluripotent stem cells as well as their ability to generate different cell types. However, it was not clear which phase PRC2 was mostly involved in during the reprogramming procedure. It has been reported that mouse embryo deficient for Ezh2 factor cannot properly complete the early stages of development [35]. In addition, it was shown that Suz12 is crucial for the precise development of the mouse. Lacking Suz12 expression resulted in the misregulation of genes other that are developmentally important for mouse development [36]. The identification of the sites controlled by Suz12 in embryonic stem cells showed that this factor was mainly involved in the regulation of transcription factors and of development determinant genes [37]. These genes were mainly located in the regions which were enriched with H3K27me3 in the nucleosomes–interestingly the portion of these genes that are necessary for development are more highly induced during the differentiation of embryonic stem cells [37]. A comparison of blastocysts derived from fertilization with blastocysts derived from Somatic Cell Nuclear Transfer (SCNT) revealed that the inner cell mass isolated from SCNT was deficient in methylating genes important for development in comparison with the inner cell mass isolated from fertilization [38]. These abnormalities, which lead to death of the embryo, could be caused by the lower expression of PRC2 members including Eed, Ezh2, and Suz12 in SCNT-derived blastocysts compared to fertilization-derived blastocysts [38]. Their study also suggested that these factors and their generated epigenetics changes could be used as the standard to assess the quality of embryos in development [38]. Collectively, we found that the down-regulation of large parts of affected genes during the late stage of reprogramming by PRC2 members could be essential for the generation and stemness maintenance of iPSCs that are capable of responding to signaling clues during the initiation of differentiation.

More interestingly, in recent years many reports have demonstrated the role of PRC2 members in reprogramming, besides their roles in development. For example, it was shown that the Ezh2 catalytic core of PRC2, which is also a H3K27 methyl-transferase, plays a crucial role during the reprogramming of somatic cells into pluripotency states [32,33,39]. It has been shown that the expression of Ezh2 continually increased during the reprogramming of fibroblasts into a pluripotency state [9,40]. In addition, it has been well-documented that repressing the expression of this methyl-transferase leads to a significant reduction of reprogramming efficiency [9,32,39]. Furthermore, the overexpression of this factor could enhance the efficiency and numbers of reprogrammed iPSC clones [9,32]. Experiments on Eed, Jarid2, Mtf2, Suz12 and other members of PRC2 showed that down-regulation of these factors resulted in decreasing reprogramming efficiency [8,39]. Jarid2 and Mtf2 function as modulators of PRC2 which act as repressors of developmentally important genes during the conversion of somatic cells into iPSCs [8]. In another study, it was shown that H3K27me3 is significantly decreased when embryonic stem cells and trophoblast stem cells lack the expression of PRC2 member Eed [41]. In addition to the involvement of PRC2 in reprogramming, PRC2-deficient embryonic stem cells failed to convert lymphocytes into pluripotent cells [42].

It seems that the function and stability of PRC2 members are associated with the presence of other members. For example, it has been demonstrated that ES cells which are deficient for the Eed factor, also have dramatically lower levels of the Ezh2 protein [41]. Our results showed that all members of PRC2 performed a role in down-regulating lineage-specific genes in the form of a complex during the maturation phase of reprogramming (Fig 1C). The expression of these factors has been previously studied in a time course study in humans, but highlights the problem in which there have been no reports that compare the time course expression of PRC2 members between mice and humans [43]. Our comparison based on the expression profiles of six independent studies showed that the expression of three members of PRC2, Ezh2, Jarid2, and Mtf2, were highly correlated in both mouse and human data sets during the maturation stage of reprogramming. Our results showed that these factors were highly expressed in both human and mouse iPSCs.

Although PRC2’s role in reprogramming had been previously described, their major regulatory role in controlling gene expression during the two transition phases of reprogramming had yet to be determined. We found that the maturation stage is much more complex than the initiation stage and less variable in different data sets compared to the initiation stage, which showed more variation even in single cell analysis [32]. As we showed, two groups of regulators are involved in this transition, with the major group of genes being involved in the methylation of H3K27. PRC2 members which are involved in the down-regulation of gene expression program (Fig 3A), are mainly regulated by pluripotency factors, including Oct4, Nanog, Sox2, and Sall4. The Oct4, Nanog, and Sox2 genes co-regulate the expression of many genes in both mouse and human pluripotent stem cells [44,45]. The core regulatory network of pluripotency genes, in addition to controlling the expression of each other, increased the expression of genes involved in stem cells characteristics [44,45]. In addition to these factors, Sall4 plays an important role in the maintenance of pluripotency properties in a self-controlling network with Oct4, Nanog, and Sox2 [46]. Here we also showed that Sall4 binds to promoters of Oct4 and Sox2 and regulates their expression [46]. It has been shown that pluripotency factors which could maintain the stemness status of iPSCs could also repress the expression of differentiation-inducing genes [44,45]. In addition, regulatory networks constructed based on ChIP binding site data for pluripotency factors Oct4, Nanog, Sox2, Sall4, and Myc, as well as PRC2 members, Ezh2, Eed, Jarid2, Mtf2, and Suz12, showed that PRC2 was significantly controlled by pluripotency factors (Fig 2E and 2F). Therefore, we concluded that during the maturation phase of reprogramming, pluripotency factors, through the induction of the expression of PRC2 complex members, could silence the lineage-specific gene expression program and maintain the ground state of pluripotency in human and mouse iPSCs.

## Supporting Information

S1 Fig Out-degree analysis of TFs during reprogramming maturation of mouse fibroblasts. (TIF)

S2 Fig Second protein complex based on valid protein-protein interactions network analysis during the maturation phase of reprogramming maturation of mouse fibroblasts. (TIF)

S1 Table List of DEGs during initiation of mouse fibroblasts reprogramming. (XLSX)

S2 Table List of DEGs during maturation of mouse fibroblasts reprogramming into iPSCs. (XLSX)

S3 Table TF-binding sites and protein-protein interactions of constructed regulatory network during maturation of mouse fibroblasts into iPSCs. (XLSX)

S4 Table Common target genes of Oct4, Sox2, and Nanog during maturation of mouse fibroblasts reprogramming into iPSCs. (XLSX)
