# Supplementary figures and images for: Involvement of Polycomb Repressive Complex 2 in Maturation of Induced Pluripotent Stem Cells during Reprogramming of Mouse and Human Fibroblasts
Source: PLoS One. 2016 Mar 3;11(3):e0150518. doi: 10.1371/journal.pone.0150518 (PMC4777544; doi:10.1371/journal.pone.0150518)

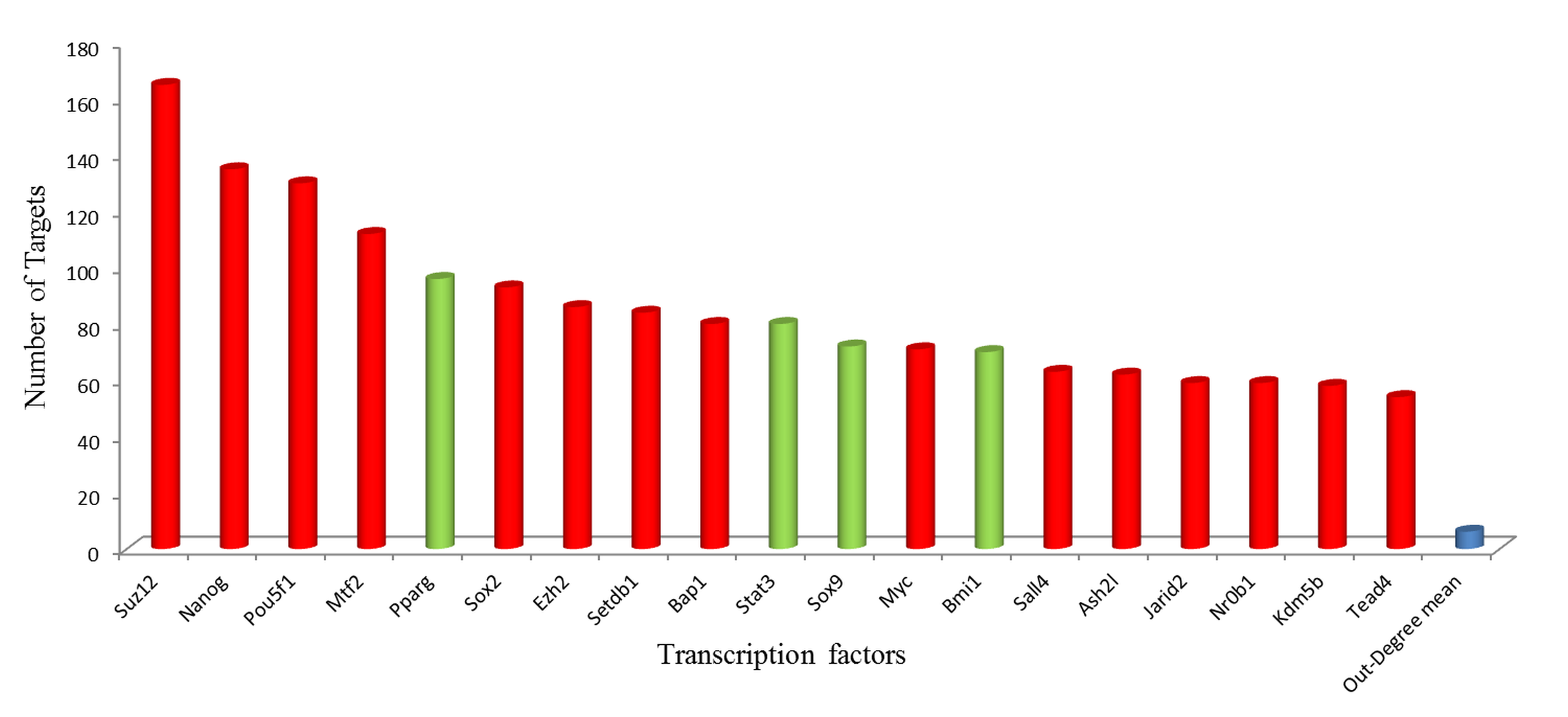

Supplement: S1 Fig — (TIF) [file pone.0150518.s001.tif]

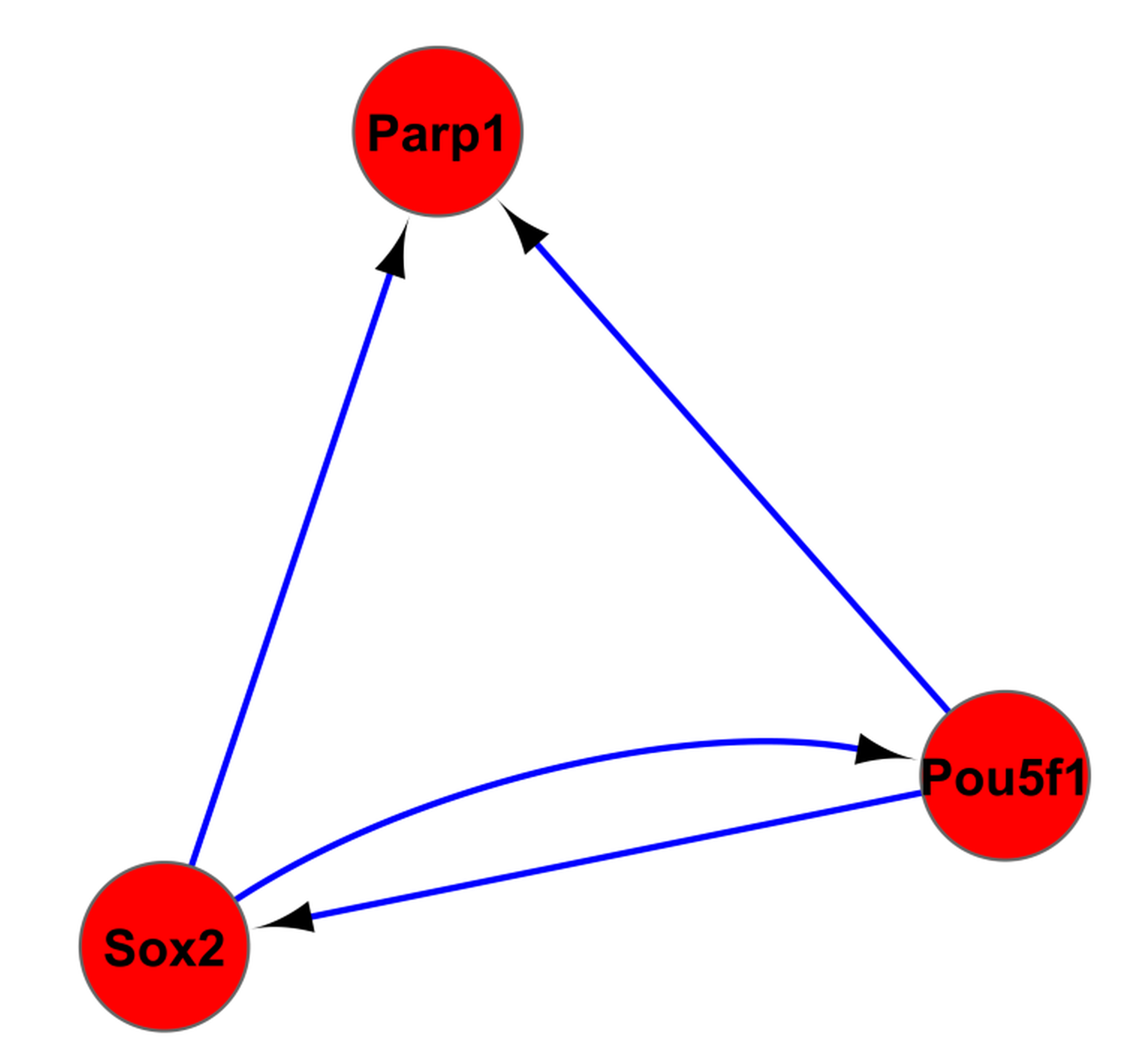

Supplement: S2 Fig — (TIF) [file pone.0150518.s002.tif]
